# Microbial community analyses of produced waters from high‐temperature oil reservoirs reveal unexpected similarity between geographically distant oil reservoirs

**DOI:** 10.1111/1751-7915.13281

**Published:** 2018-05-27

**Authors:** Daehyun D. Kim, Corynne O'Farrell, Courtney R. A. Toth, Oscar Montoya, Lisa M. Gieg, Tae‐Hyuk Kwon, Sukhwan Yoon

**Affiliations:** ^1^ Department of Civil and Environmental Engineering KAIST Daejeon Korea; ^2^ Department of Biological Sciences University of Calgary Calgary AB Canada

## Abstract

As a preliminary investigation for the development of microbial‐enhanced oil recovery strategies for high‐temperature oil reservoirs (~70 to 90°C), we have investigated the indigenous microbial community compositions of produced waters from five different high‐temperature oil reservoirs near Segno, Texas, U.S. (~80 to 85°C) and Crossfield, Alberta, Canada (~75°C). The DNA extracted from these low‐biomass‐produced water samples were analysed with MiSeq amplicon sequencing of partial 16S rRNA genes. These sequences were analysed along with additional sequence data sets available from existing databases. Despite the geographical distance and difference in the physicochemical properties, the microbial compositions of the Segno and Crossfield produced waters exhibited unexpectedly high similarity, as indicated by the results of beta diversity analyses. The major operational taxonomic units included acetoclastic and hydrogenotrophic methanogens (*Methanosaetaceae*,* Methanobacterium* and *Methanoculleus*), as well as bacteria belonging to the families *Clostridiaceae* and *Thermotogaceae*, which have been recognized to include thermophilic, thermotolerant, and/or spore‐forming subtaxa. The sequence data retrieved from the databases exhibited different clustering patterns, as the communities from close geographical locations invariably had low beta diversity and the physicochemical properties and conditions of the reservoirs apparently did not have a substantial role in shaping of microbial communities.

## Introduction

Petroleum remains as the primary source of energy, in spite of the recent surge in energy production from alternative, renewable sources (Sieminski, 2015). The total global reserve of petroleum is by no means scarce; however, the deposits of easily recoverable crude oil that can be put into production using conventional production technology are dwindling. Most of potential deposits of conventional oil have already been explored and remaining unexplored deposits often pose challenges for economical production due to their remoteness or environmental sensitiveness (Muggeridge *et al*., 2014). To enhance the recovery efficiencies of the developed oilfields, various enhanced oil recovery (EOR) techniques, for example injection of miscible gas (e.g., CO_2_ and N_2_), steam flooding and injection of polymers and/or surfactants, have been developed and implemented (Youssef *et al*., 2009). Another EOR technique that has gathered significant interest is the microbial‐enhanced oil recovery (MEOR) technique, wherein microbial activities are utilized to partially degrade long‐chain hydrocarbons, produce gases and organic acids, and/or alter wettability of solid surfaces, all aiming to enhance oil mobility (Jack, 1991; Youssef *et al*., 2009).

Although MEOR is an attractive energy‐efficient and environmentally friendly alternative, it has not been widely implemented. One of the barriers to broad implementation of MEOR practices is the paucity of reliable data from scientific investigations that can be used to predict behaviour of the indigenous microbial community upon *in situ* biostimulation or bioaugmentation (Youssef *et al*., 2009; Lewin *et al*., 2014). Since the 1980s, scientific investigations, both culture‐based and culture‐independent, have been performed to better understand the microbial community structures and metabolic potentials in developed oil reservoirs (Mueller and Nielsen, 1996; Orphan *et al*., 2000, 2003). More recently, high‐throughput sequencing techniques (e.g., pyrosequencing and Illumina HiSeq/MiSeq sequencing technologies) have been implemented to analyse the microbial community compositions and metagenomes of oil reservoirs (An *et al*., 2013; Lewin *et al*., 2014; Frank *et al*., 2016; Hu *et al*., 2016; Vigneron *et al*., 2017). These investigations have demonstrated that stable active microbial populations are established in the subsurface oil reservoirs with temperatures up to 80°C and even higher, and also that anthropogenic alterations, for example injection of nitrate to treat souring, may lead to perturbation of the indigenous microbial populations and their metabolic properties. Nevertheless, the data acquired in previous research projects are yet too quantitatively limited to cover the diverse environmental conditions that can characterize subsurface oil reservoirs. Due primarily to the difficulty in sample collection and limited public disclosure of information from the oil industry sector, the availability of sequencing data in public databases remains limited.

In this study, the microbial community compositions in produced waters collected from four separate high‐temperature oil wells in the Segno oilfield near Houston, TX, USA (80–85°C) and an oil well in the Crossfield oilfield, AB, Canada (75°C) were analysed to enhance the understanding of the microbial community structure in the deep subsurface high‐temperature (~80°C) oil reservoirs, as a preliminary investigation for developing MEOR strategies for these types of reservoirs. Microbial community compositions of such high‐temperature reservoirs have rarely been investigated. Furthermore, we analysed these microbial community profiles alongside those from oil reservoirs (produced water samples) across the globe having diverse physicochemical properties. These investigations revealed an unexpected level of similarity among the microbial communities of the high‐temperature oil reservoirs in two geographically distant locations that have not yet been observed in oil reservoir microbiomes.

## Results and discussion

### The physicochemical properties of the produced water samples

The physicochemical properties of the produced water samples (CFW: Crossfield well sample; CFS: Crossfield separator sample; SG30, SG54, SG80, and SG85: Segno well samples from wells 30, 54, 80, and 85, respectively) were analysed before sequencing (Table S1). The pH of the four produced water samples from Segno oil fields varied from 5.53 to 8.03. The produced water samples from oil well at the Crossfield site was weakly acidic (pH 4.70), while the produced water collected from the separator was measured to be pH 7.33. All of the produced water samples contained relatively high concentrations of organic acids including acetate, propionate, and formate, which may serve as labile electron donors for indigenous microbial population. The produced water from the oil well at the Crossfield site (CFW) had higher concentrations of ${\text{SO}}_{4}^{2-}$ (23.7 mg l^‐1^) compared to the four production water samples from Segno site (3.3–12.7 mg l^‐1^). The ${\text{NO}}_{3}^{-}$ concentrations in all produced water samples fell below the limit of detection (<1 mg l^‐1^), suggesting ${\text{NO}}_{3}^{-}$ respiration is unlikely. Ferric iron was detected only from SG30 at 4.8 mg l^‐1^; however, ferrous iron was detected in three of the four Segno samples (46.5–80.5 mg l^‐1^) and the Crossfield (13.9 mg l^‐1^ sample) at substantially larger concentrations. The low concentrations of these potential electron acceptors suggested that the microbiomes of these subsurface oil reservoirs may be electron acceptor‐limited. Phosphate (${\text{PO}}_{4}^{3-}$) concentrations were below the detection limit (1 μg l^‐1^) in the produced water samples from both Segno and Crossfield sites, suggesting that lack of bioavailable phosphorous may also limit microbial growth *in situ*. The qPCR assays targeting universal 16S rRNA genes demonstrated that the microbial populations in the produced water samples, 172 ± 52 to 489 ± 178 copies (mL produced water)^−1^, were orders of magnitudes lower than the cell numbers observed in produced waters from previously investigated oil reservoirs with comparable temperature ranges (Gittel *et al*., 2009; Li *et al*., 2017). Such low microbial cell counts were previously reported only in hyperarid Atacama soils and high Arctic permafrost samples, both of which are hostile to microbial habitation (Yergeau *et al*., 2010; Fletcher *et al*., 2011).

### Microbial community profiles in the produced water samples

**Figure 1:**
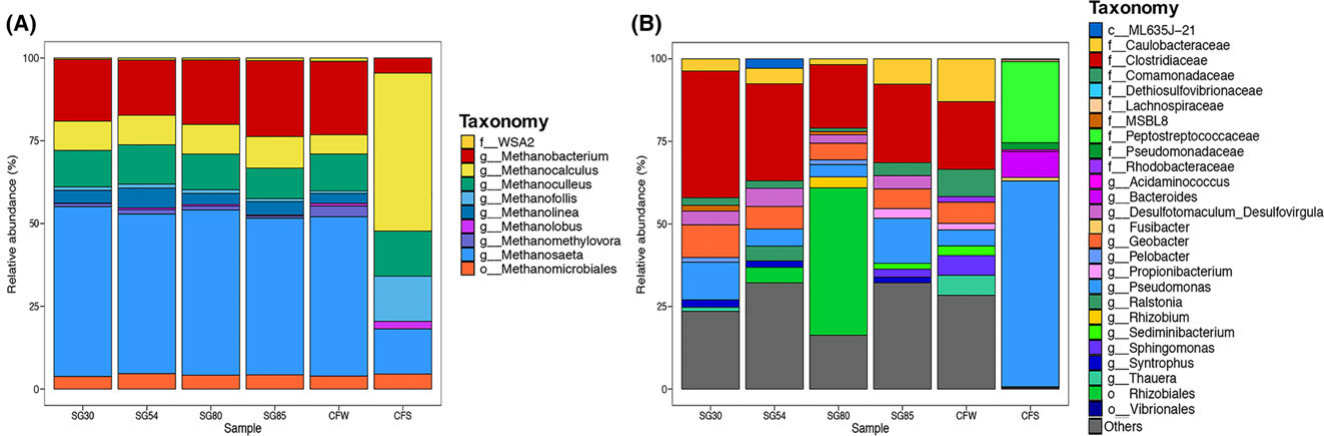
The compositions of (A) archaeal and (B) bacterial population in produced waters from oil reservoirs near Segno, Texas (SG30, SG54, SG80, SG85) and Crossfield, Alberta (CFW, CFS). The notations in the legend f, c, o, and g stand for the OTUs assigned to family, class, order and genus levels respectively. The processed reads were clustered into OTUs by assigning to the Greengenes v13.8 reference database or *de novo* clustering (for reads with no matches in the database) with a cut‐off value of 97%.

The microbial community compositions of the produced water samples from Crossfield and Segno were analysed with 16S rRNA amplicon sequencing using an Illumina MiSeq sequencing platform. After quality screening and merging of the paired‐end sequences, amplicon sequencing of the produced water samples yielded an average of 65 230 reads (45 944–103 164 reads) per sample (Table S2). The Good's coverage indices of >99% and saturated rarefaction curves (Fig. S1) indicated that most microbial diversities in the samples were covered with the 16S amplicon sequencing analyses. The Shannon‐Wiener indices of the samples ranged from 5.202 to 6.689, while the index value for the Crossfield separator sample was 2.370. The microbial community profiles of the produced water collected from the oil wells were strikingly similar considering the geographical distance between Segno and Crossfield oil fields and the differing chemical compositions; however, the microbial community composition of the Crossfield separator sample deviated greatly from the produced water collected from the oil well at the same site with relatively similar chemical composition (Fig. 1).

Archaea constituted 18.6–33.4% of the total microbial population in the produced water samples. In all produced water samples, >90% of the archaeal OTUs were affiliated to organismal groups identified as methanogens. The archaeal population profiles were virtually indistinguishable among the produced water samples from the oil wells, with *Methanosaeta*, an acetoclastic methanogen, as the most abundant archaeal genus (45.9–49.8% of archaeal population) in each of the produced water samples. Hydrogenotrophic (*Methanobacterium*,* Methanocalculus*,* Methanoculleus, Methanofollis, Candidatus Methanoregula,* and *Methanolinea*: 42.5–47.2% of the archaeal populations) and methylotrophic methanogens (*Methanolobus* and *Methanomethylovorans*: 1.0–3.7% of the archaeal populations) were also found in the produced water samples, suggesting that multiple methanogenic pathways may coexist in the examined oil reservoirs despite the presence of dissolved ${\text{SO}}_{4}^{2-}$ and presumed sulphate reduction activities that may inhibit methanogenesis (Fig. 1A). Uncharacterized WSA2 class (phylum *Euryarchaeota*) was also recovered, although at much lower relative abundances (0.7–1.4%).

The OTUs affiliated to members of the classes *Clostridia* and *Alphaproteobacteria* dominated the bacterial populations in the production water samples from the oil wells. *Clostridia* constituted the most abundant taxa (of all bacteria and archaea) in the samples CFW, SG30, SG54, and SG85, while members of the *Alphaproteobacteria* were dominant in the sample SG80 (Fig. 1B). Members of *Clostridia* are known to thrive in moderately thermophilic regimes and are known to be spore‐forming, and thus, may be suited to survive amid shifting temperature and pressure conditions (de Rezende *et al*., 2012; Aüllo *et al*., 2013). Within the *Clostridia* class, only 14.6–20.9% of OTUs could be identified at the genus level and 38.7% of these OTUs were identified as *Desulfotomaculum*, a genus generally known as thermophilic sulphate‐reducing bacteria (Table S3) (Aüllo *et al*., 2013). Several isolates and enrichments of *Desulfotomaculum* spp. have also been found to form syntrophic associations with hydrogenotrophic methanogens, which may be one of the pathways contributing to CH_4_ production in the examined oil reservoirs (Imachi *et al*., 2006). *Alphaproteobacteria* class was also abundant in the produced water samples. Especially in the CFW sample, a member of *Alphaproteobacteria* (assigned to the order *Rhizobiales*) was the most abundant group of microorganisms*,* accounting for 37.7% of the total microbial population (Table S3). The order *Rhizobiales*, members of which are known as anaerobic hydrocarbon degraders, was previously identified as one of the major groups of organisms inhabiting several thermophilic (55–70°C) Chinese oil reservoirs (Zhang *et al*., 2012).

Apart from *Clostridia* and *Alphaproteobacteria*,* Deltaproteobacteria* also constituted a substantial proportion of the produced water samples (8.9‐16.6% of the total bacterial OTUs) (Fig. 1B). The majority of the OTUs affiliated to *Deltaproteobacteria* (54.7‐63.8% of *Deltaproteobacteria* and 4.2‐6.5% of total bacterial OTUs) were identified to be affiliated to *Geobacter*, a genus well‐known for its metal‐reducing metabolic capabilities. The presence of *Geobacter* spp. was previously observed in a mid‐temperature oil reservoir (59°C) in Dagang, China (Nazina *et al*., 2017). Fe^3+^, the typical substrate for metal‐reducing *Geobacter* spp., was absent in most of the produced water samples; however, substantial concentrations of Fe^2+^ in produced water samples suggest that iron reduction may have been coupled to abiotic/biotic iron oxidation taking place *in situ*. As *Geobacter* spp. are known to utilize a broad range of electron acceptors, the possibility that *Geobacter* spp. in the oil reservoirs may utilize other electron acceptors should not be dismissed (Sung *et al*., 2006). Several members of the *Geobacter* genus (e.g., *G. metallireducens* and *G. sulfurreducens*) were previously found to be capable of degrading aromatic hydrocarbons and thus, may break down complex hydrocarbons in the oil reservoirs (Coates *et al*., 1997; Zhang *et al*., 2010). The *Deltaproteobacterial* OTUs recovered from the oil wells also included syntrophic bacteria, *Pelobacter* spp. and *Syntrophus* spp., presumably involved in methanogenic hydrocarbon biodegradation *in situ* in association with methanogens (Gray *et al*., 2011).

### Comparative analyses of oil reservoir microbial communities

**Figure 2:**
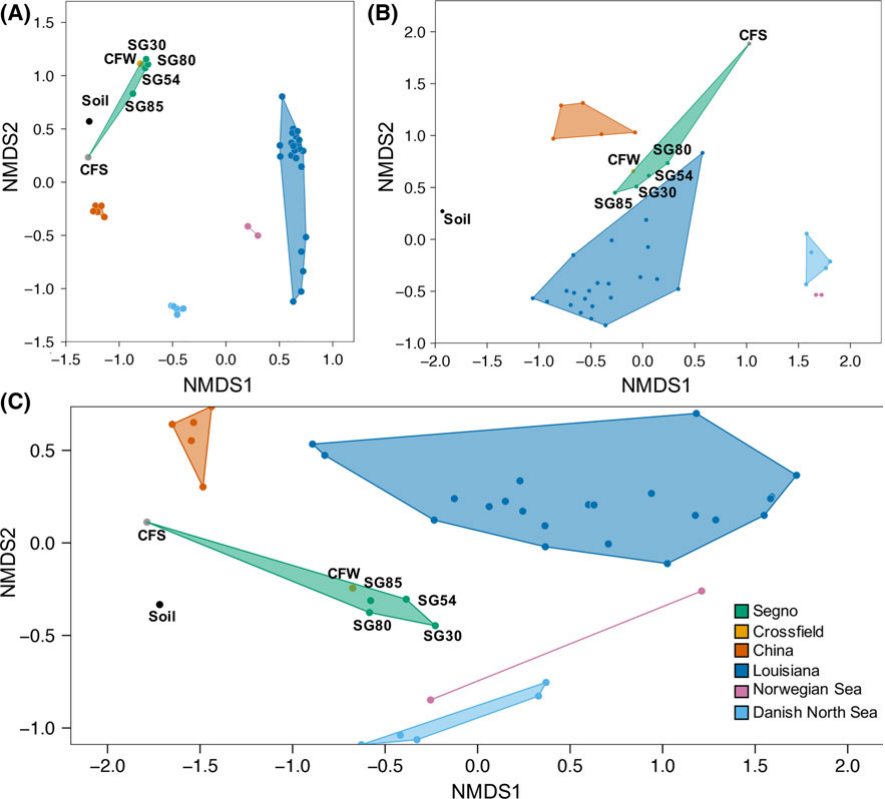
The Nonmetric multidimentional scaling (NMDS) plot of the produced water microbial communities reconstructed from the 16S rRNA sequence data sets acquired experimentally in this study and from databases. Three different metrics were used for beta diversity calculation: (A) Bray‐Curtis distance metric, (B) unweighted Unifrac, (C) weighted Unifrac. The colour shades denote the communities originating from the same geographical regions as indicated in the legend. The stress values for (A), (B) and (C) are 0.213, 0.140 and 0.089 respectively. The microbial community of an agricultural soil sample was plotted as an out‐group (accession number: PRJNA430535).

The microbial community profiles of the Crossfield and Segno produced waters were analysed in depth alongside the microbial community profiles of produced waters from other oil reservoirs of varying geographical locations and physicochemical characteristics (Table S4). Comparisons were constructed with the raw 16S rRNA amplicon sequence data sets acquired from the NCBI Sequence Read Archive (SRA) and through personal communication (Lewin *et al*., 2014; Gao *et al*., 2015; Hu *et al*., 2016; Shelton *et al*., 2016; Li *et al*., 2017; Vigneron *et al*., 2017). As expected from the nearly identical microbial compositions of the Crossfield and Segno samples, tight clustering was observed for the microbial communities in these samples, regardless of the metrics used for beta diversity calculation (Fig. 2). Except for the Segno and Crossfield produced water communities, no obvious clustering of the communities from produced waters collected from distant geographical regions was observed. The two produced water samples from the separate oil reservoir formations (~3 km apart) in the Norwegian Sea, previously reported to exhibit unusual similarity (Lewin *et al*., 2014), clustered closely when analysed with Bray‐Curtis or unweighted Unifrac distance metrics, but were distantly plotted with weighted Unifrac distance metrics. The only instance of tight clustering between communities constructed with data sets from two independent studies was observed between the communities from the oil wells in the Norwegian Sea and Halfdan oil field in the Danish North Sea, albeit only when analysed with unweighted Unifrac distance metric. This close phylogenetic association between the OTUs in these samples may still be attributed to their geographical proximity (Fig. S2) (Lewin *et al*., 2014; Vigneron *et al*., 2017). The physicochemical characteristics of the tightly‐clustered oil reservoirs exhibited little consistency (Table S4) and the clustering patterns appeared to depend primarily on the geographical proximity, rather than any specific physicochemical parameter. The close association of the produced water microbial communities in Segno and Crossfield sites, two geographical locations separated by 2850 km (1771 miles) of discontinuous terrain, was thus a unique observation.

**Figure 3:**
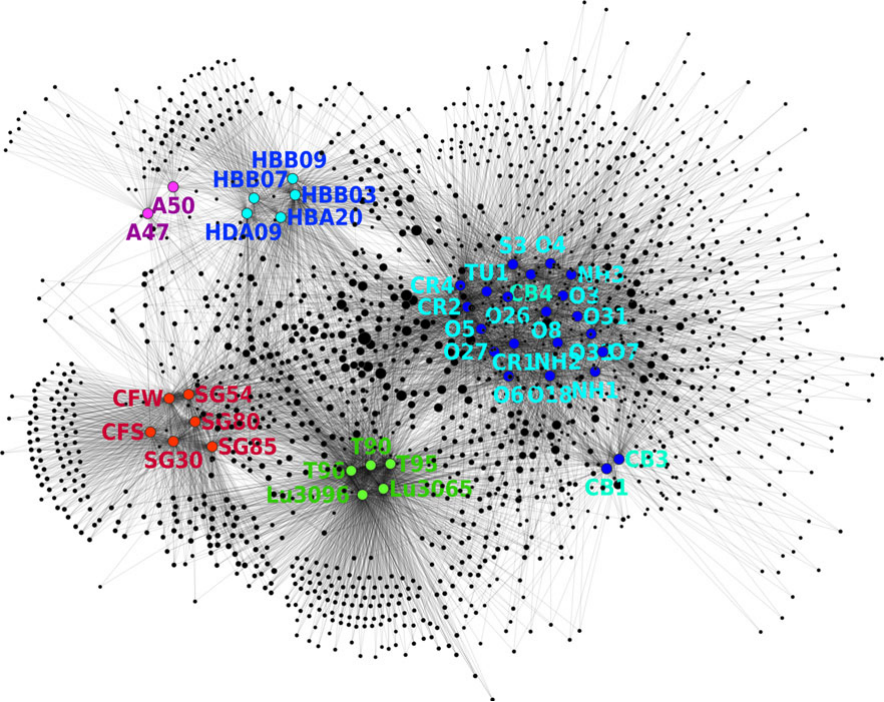
The OTU network map showing the sharing of OTUs among the produced water microbial communities. The black nodes represent the OTUs generated from the sequence data sets. The OTUs with abundance larger than 0.1% in at least one of the samples were included in the analysis. The coloured nodes represent the produced water samples node and are labelled with the sample ID's. (Segno and Crossfield: red, Xinjiang, China: green, Louisiana: blue, Norwegian Sea: pink, Danish North Sea: sky blue). The edge connection between an OTU node and a sample node denote the occurrence of the OTU in the sample at >0.1% relative abundance. The size of each OTU node is proportional to the number of the samples where the corresponding OTU was recovered in relative abundance larger than 0.1%.

An OTU network analysis was performed to observe the clustering patterns of the produced water communities based on sharing of OTUs among the communities. The clustering of OTUs in the centre of the field indicated that a large number of microbial taxa were shared by the produced water communities; however, formations of distinct clusters among the communities were also evident in the network. Sharing of unique OTUs resulted in tight clustering of the communities from close geographical locations, corroborating the result from the nonmetric multidimensional scaling (NMDS) plots. Such dependence on the geographical distances is clearly distinct from what is observed in soils and aquatic environments with more populated microbiomes, and this difference may be attributed to the extremely slow generation time of the microorganisms residing in the adverse subsurface environments (Lozupone and Knight, 2005; Chu *et al*., 2010; Lewin *et al*., 2014). Once again, all of the SG and CF communities were clustered within a single tight group, while this group was clearly separated from any other cluster. Also, notable in this analysis was that the CFS community clustered tightly with the CFW and SG communities, sharing a large number of the OTUs unique to these communities, supporting that the microorganisms in the CFS community originated from the CFW community (Fig. 3).

Cross contamination of the produced water samples or extracted DNA may be raised as a possibility; however, several lines of evidence arguing against this possibility can be found in the experimental results. (i) The produced water samples from the Segno and Crossfield sites were sampled and processed separately on different dates and sequenced in separate MiSeq sequencing runs. (ii) A close association was observed between the CFW and CFS communities on the OTU network, indicating that the CFS microbial community originated from the CFW community. The separator DNA sample was less susceptible to contamination with Segno DNA samples due to its high DNA concentration and thus, the resemblance of OTU composition between CFS and the CFW provides an indirect evidence that the similarity between SG and CFW communities was not due to cross contamination. (iii) Contamination of the SG DNA samples with the CFW DNA sample was unlikely, as the four Segno microbial communities exhibited very similar microbial compositions, which are distinguishable from that of the CFW microbial community (Fig. 1). The similarity of the CFS community constructed with the 16S rRNA sequences in the shotgun metagenome to that constructed with amplicon sequences precludes another possibility that PCR bias in amplicon sequencing may have been the cause (Fig. S3).

The microbial communities in the produced waters from the Halfdan oil field in the North Sea, with reservoir temperatures similar to the Segno and Crossfield sites (73–76°C), were plotted distantly from the SG and CFW communities on any of the NMDS plots and in the OTU network analysis. With limited information, it is not yet possible to identify the rationale for the divergence among these high‐temperature reservoirs. Despite these differences, one common observation that can be made of these microbial communities from the high‐temperature oil reservoirs is that members of the class *Clostridia* were among the most abundant taxa in these communities. The abundance of the organisms affiliated to the *Clostridia* class in these high‐temperature oil reservoirs was also consistent with previous reports that this group of Gram‐positive bacteria often constitute major portions of the microbial populations in deep subsurface environments stressed with high temperature and high pressure (Gittel *et al*., 2009; Aüllo *et al*., 2013; Frank *et al*., 2016). This relative abundance was attributed to the spore‐forming capability that may have helped these organisms withstand the shifting environmental conditions during the long sedimentation and burial processes of the geologic formations, as well as their capability to survive under high temperature and pressure (Aüllo *et al*., 2013).

### Co‐occurrences of key oil reservoir microbial taxa

**Figure 4:**
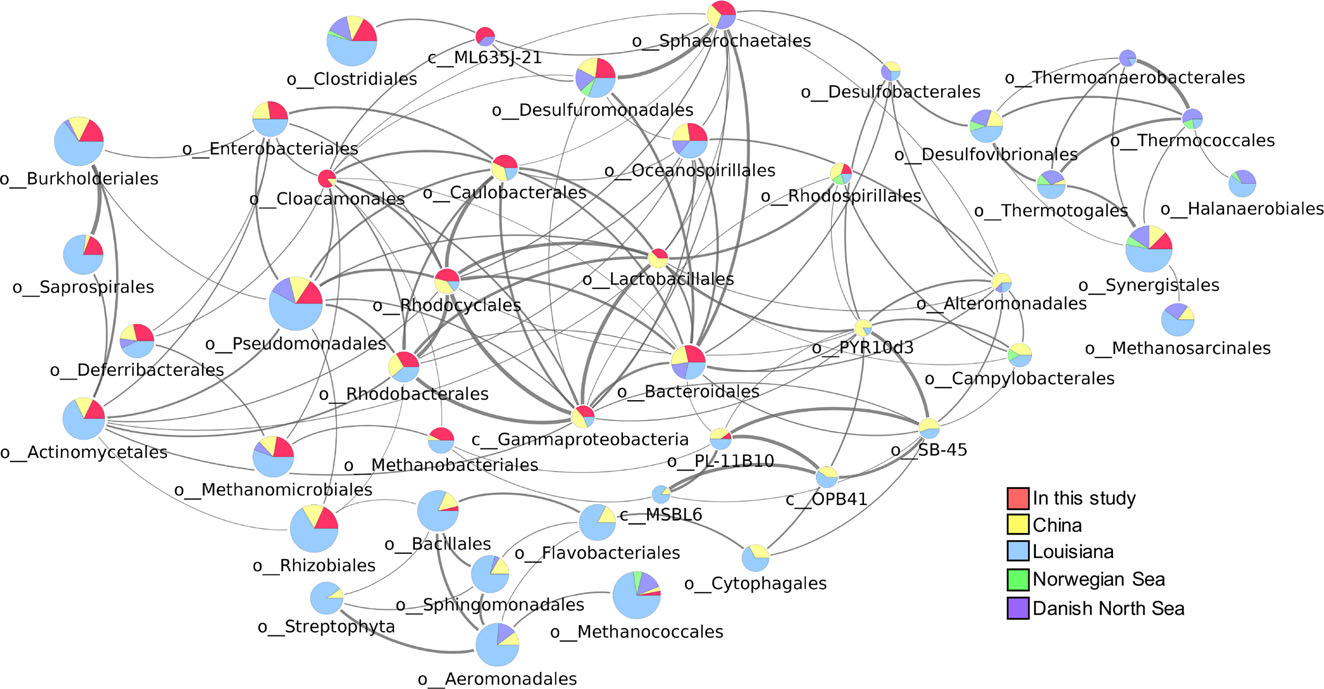
Co‐occurrence network of the microbial taxa recovered from the produced water samples. The OTUs with >1% relative abundance in any of the samples were assigned to the order level (or class level for the OTUs that could not be assigned to a specific order) and represented as nodes in the network. The Spearman's coefficient >0.5 was regarded as positive relationship between the taxa. Width of an edge connecting two taxa is proportional to the Spearman's coefficient, with thicker edge indicating a stronger positive co‐occurrence relationship. The size of a node is proportional to the number of samples where the taxa was recovered at >1% relative abundance. Each node is represented as a pie graph illustrating the relative contributions of the different oil fields. The taxa recovered exclusively in produced water samples from a single oil field site were excluded from the analysis.

To check for potential relationships among key microbial taxa in the oil reservoir microbiomes, co‐occurrence patterns of major microbial taxa (microbial orders found at >1% abundance in at least one sample) were analysed and presented on a co‐occurrence network (Fig. 4). The microbial taxa that appeared as major populations in the largest number of data sets, *i.e*., *Clostridiales* (in 35 data sets), *Pseudomonadales* (in 38 data sets), *Burkholderiales* (in 34 data sets), *Rhizobiales* (in 33 data sets), *Methanococcales* (in 33 data sets), and *Synergistales* (in 32 data sets), were positioned in the periphery of the co‐occurrence network, suggesting that these taxa have little reliance on other specific taxa of organisms for colonization. Most notable was *Clostridiales*, one of the most abundant groups of microorganisms in the high‐temperature oil reservoirs including the Segno and Crossfield sites, in that virtually no significant co‐occurrence relationship was found with any group of microorganisms other than the weak correlation with uncultured *Cyanobacteria* sp. ML635J‐21. A strong co‐occurrence relationship was observed among the orders *Sphaerochaetales*,* Desulforomonadales*,* Oceanospirillales*, and *Bacteroidales*, which are all known to harbour taxa that are obligately anaerobic hydrocarbon‐utilizing organisms, suggesting that the anoxic, hydrocarbon‐rich oil reservoirs favour colonization of these obligate anaerobes. The co‐occurrence analysis also suggests that these organisms are not likely to compete for a common limiting substrate (more likely to be electron acceptor or nutrients, as supple hydrocarbon availability is guaranteed in the oil reservoirs) and possibly share a symbiotic or syntrophic relationship (Head *et al*., 2014). All of these taxa were relatively abundant in the Segno and Crossfield samples and are likely to contain thermophilic and barotolerant subgroups able to survive in such adverse subsurface environments.

### Implications for MEOR

As suggested by low cell counts, the examined high‐temperature oil reservoirs appear to be hostile to microbial habitation despite the abundance of organic materials as the potential source of carbon and energy. Developing suitable MEOR techniques targeting such reservoirs would be no easy undertaking, due to the difficulty in establishing a viable population of metabolically active microorganisms able to withstand these adversities. Paradoxically, the scantiness of established microbial population may also work in favour of MEOR as the success of MEOR often depends primarily on the competition of these externally introduced organisms with indigenous microorganisms (Gray *et al*., 2008; Youssef *et al*., 2009). Successful enrichment of useful microorganisms is not guaranteed from such sparse inoculum; however, the similarity observed between microbiomes of the high‐ temperature produced water samples and the relative abundance of the previously enriched microbial taxa, *e.g*., *Chlostridia* and methanogens, are certainly positive signs. The high temperature, *per se*, should not be the adverse environmental parameter limiting the microbiome to such low population. The availability of readily utilizable organic electron donors *in situ* suggests against the possibility of electron donor starvation. Enrichment efforts should thus focus on finding electron acceptors or nutrients that would stimulate microbial growth, as potential electron acceptors (e.g., Fe^3+^ and ${\text{NO}}_{3}^{-}$) and nutrients (e.g., ${\text{PO}}_{4}^{-}$) were deficient in the examined samples. The decision as to which mechanism should be the major target for development of MEOR technology for such high‐temperature reservoirs awaits the success of these initial enrichment efforts.

## Conflict of interest

The authors declare no conflict of interest.

## Supporting information


**Fig. S1.** Rarefaction curves of the OTUs recovered from the produced water samples.
**Fig. S2.** Geographical locations of the oil well sites where the produced water samples were collected for microbial community analyses.
**Fig. S3.** Comparison of microbial community profiles of the CFS sample constructed with 16S amplicon sequencing and shotgun metagenome sequencing.
**Table S1.** Physiochemical characterization of the produced water samples from Segno and Crossfield oil wells.
**Table S2.** Summary of 16S rRNA amplicon sequencing of the produced water samples.
**Table S3.** Relative abundance of taxa found in the produced water samples from Segno and Crossfield.
**Table S4.** Imported physicochemical characterization data for the produced water samples whose microbial communities were analysed in this study.
**Table S5.** The OTU table of produced water samples from Segno and Crossfield sites including the sample collected from the oil‐water separator.
**Appendix S1.** Materials and methods.
**Appendix S2.** Supplementary results.
