# Supplementary material for: Microbial community analyses of produced waters from high‐temperature oil reservoirs reveal unexpected similarity between geographically distant oil reservoirs
Source: Microb Biotechnol. 2018 May 27;11(4):788–96. doi: 10.1111/1751-7915.13281 (PMC6011920; doi:10.1111/1751-7915.13281)
Supplement: Supplementary file 1 — Fig. S1. Rarefaction curves of the OTUs recovered from the produced water samples. Fig. S2. Geographical locations of the oil well sites where the produced water samples were collected for microbial community analyses. Fig. S3. Comparison of microbial community profiles of the CFS sample constructed with 16S amplicon sequencing and shotgun metagenome sequencing. Table S1. Physiochemical characterization of the produced water samples from Segno and Crossfield oil wells. Table S2. Summary of 16S rRNA amplicon sequencing of the produced water samples. Table S3. Relative abundance of taxa found in the produced water samples from Segno and Crossfield. Table S4. Imported physicochemical characterization data for the produced water samples whose microbial communities were analysed in this study. Table S5. The OTU table of produced water samples from Segno and Crossfield sites including the sample collected from the oil‐water separator. Appendix S1. Materials and methods. Appendix S2. Supplementary results. [file MBT2-11-788-s001.docx]

**Supporting Information**

**Methods and materials**

**Sample collection.**

The production water samples obtained from the oilfield near Segno were collected directly from the wellheads on Feb. 25, 2016. Sampling at the Crossfield oilfield was conducted on Jul. 13, 2016. The produced water samples from the wellheads contained a floating layer of oil, while the water sample collected from the oil-water separator consisted mainly of a single aqueous phase. The samples were collected in pre-sterilized 500-mL Nalgene square media bottles (Thermo Fisher Scientific, Waltham, MA). The bottles were filled up to the brim and sealed to minimize oxygen ingress. The sample bottles were immediately placed in a cooler (maintained below 4°C) and transported to the University of Calgary, where the samples were processed for microbial community analysis. The samples were stored at 4°C until further treatment.

**Analytical methods for characterization of the produced water samples**

The collected produced water samples were analyzed for the physicochemical properties (pH, salinity and concentrations of organic acids, anions and ferric/ferrous iron) that may be relevant to indigenous microbial communities. The salinity of the samples was measured with Vernier conductivity probe (Vernier Software & Technology, Beaverton, OR). The concentrations of organic acids were measured using a LC-20A series high-performance liquid chromatograph (Shimadzu Corporation, Kyoto, Japan) equipped with a HPX-87H column (Bio-Rad Laboratories, Hercules, CA) and a photodiode array detector. The concentrations of relevant anions (Cl^-^, NO_2_^-^, NO_3_^-^, PO_4_^3-^ and SO_4_^2-^) in the produced water samples were measured with Metrohm 883 BASIC IC Plus ion chromatograph equipped with Metrosep A Supp 4-250/4.0 column (Metrohm, Riverview, FL). The samples were diluted ten-fold with distilled water before each measurement to remove noise arising from impurities in the matrices. The dissolved ferric and ferrous iron concentrations in the produced water samples were measured with the ferrozine method (Viollier et al., 2000). Analyte concentrations were determined by comparison with calibration curves constructed from standards.

The total microbial population in the samples were quantified using quantitative polymerase chain reactions (qPCR). Three-milliliter aliquots of aqueous portions of produced water samples were centrifuged at 18,400xg and DNA was extracted from the pellets using DNeasy Blood & Tissue Kit (QIAGEN, Hilden, Germany) according to the protocol provided by the manufacturer. The qPCR enumeration of bacterial population was performed with QuantStudio 3 real-time PCR system (Thermo Fisher Scientific, MA, USA) using TaqMan detection chemistry (Applied Biosystems, CA, USA). The primers/probe set targeting eubacterial 16S rRNA genes, 1055f (5’-ATGGCTGTCGTCAGCT-3’) and 1392r (5’- ACGGGCGGTGTGTAC-3’) / Bac1115Probe (5’-CAACGAGCGCAACCC-3’), was used for amplification (Ritalahti et al., 2006).

**DNA extraction and 16S rRNA amplicon sequencing**

DNA was extracted from the produced water samples for analyses of microbial community compositions. Due to the extremely low biomass concentrations, the produced water samples required rigorous DNA extraction procedures. For the Segno samples, DNA was collected from both oil and aqueous layers. The layers of oil and oil-water interface were carefully pipetted off and stored separately. The aqueous fraction (300 mL) was vacuum-filtered through a 0.2 μm bottle-top filter (VWR International, Radnor, PA). The four fractions (the oil fraction, the oil-water interface, the filter paper, and the unfiltered aqueous fraction) were stored at –20°C until further processing. DNA extraction was performed with FastDNA^®^ SPIN Kit for Soil DNA Extraction (MP Biomedicals, Santa Ana, CA) according to the instructions provided by the manufacturer with an additional washing step with 500 μL of 5.5 M guanidine thiocyanate solution (Alain et al., 2011). The DNA extracted from the four sample fractions were pooled and the concentration was measured with Qubit^TM^ 2.0 fluorometer (Thermo Fisher Scientific). As the concentrations of the pooled DNA samples were below the detection limit (0.5 ng/mL), the DNA samples were concentrated by reducing the volume to 20 μL using Amicon^®^ Ultra – 0.5 mL centrifugal filters (Millipore, Billerica, MA). Subsequently, PCR amplifications targeting the V6-V8 region of the 16S rRNA gene were performed using universal primers 16S-926Fi5 (5’- TCGGCAGCGTCAGATGTGTATAAGAGACAGAAACTYAAKGAATTGACGG -3’) and 16S-1392r (5’- GTCTCGTGGGCTCGGAGATGTGTATAAGAGACAGACGGGCGGTGTGTRC 3’) in 8 replicates per sample. In each PCR reaction, 2.5 μL of DNA solution was used as the template for a 25-μL reaction. A PCR protocol previously designed for acquisition of high amplicon yields from template DNA with low concentration was used for amplification (Klindworth et al., 2012). After amplification, PCR reaction solutions were combined and concentrated using the centrifugal filters.

A simpler approach was used for extraction of DNA from the Crossfield produced water samples, as the amount of DNA extractable from the oil layer appeared to be negligible and therefore DNA extraction from oil phase was deemed unnecessary. The produced water samples from the oil well and the separator at the Crossfield site (240 mL each) were separated into oil and aqueous layers by centrifuging the samples at 25,800xg for 10 min. The oil layer was then pipetted off and discarded. The remaining aqueous portion was centrifuged at 12,000xg for 20 min and the supernatant was removed. The pellets were subjected to DNA extraction using the FastDNA SPIN Kit with the additional washing step with 5.5 M guanidine thiocyanate solution. The eluents (300 μL) were concentrated to 30 μL using the centrifugal filters. After PCR amplification was performed as described above in 12 replicates, the products were combined and concentrated.

The final concentrations of the concentrated 16S rRNA PCR amplicons were sufficiently high for downstream processing (5.44 – 55 ng/μL for all Segno and Crossfield samples). The concentrated 16S rRNA amplicons were indexed with the second round of PCR reactions using the Nextera XT Index Kit (Illumina, CA). Each of the forward primers contained a 5’ adapter (5’-AATGATACGGCGACCGAGATCTACAC-3’), an 8-nt index sequence, and a forward primer overhang adapter (5’-TCGTCGGCAGCGTC-3’) attached to the 5’ end. Each of the reverse primers contained a 5’ adapter (5’-CAAGCAGAAGACGCCATACGAGAT-3’), an 8-nt primer index, and an overhang adapter (5’-GTCTCGTGGGCTCGG-3’) attached to the 5’ end. Amplifications were performed in triplicates of 50-µL reactions, which were subsequently pooled and purified using the Mag-Bind^®^ RxnPure Plus Kit (Omega Bio-Tek, Norcross, GA). The purified amplicons were quantified, normalized to 2 nM, and pooled into the library. The resulting library was verified for the expected length of DNA (~600 bp) using the High Sensitivity DNA Analysis Kit and 2100 Bioanalyzer System (Agilent Technologies, Santa Clara, CA). High-throughput sequencing of the 16S rRNA gene library was performed on an Illumina MiSeq benchtop sequencer with MiSeq Reagent Kit v3 (Illumina). As a performance control, phiX Control v3 (Illumina) was added to 5% of the total amount of DNA in the library. The raw sequence datasets acquired from this study can be found in the National Center for Biotechnology Information (NCBI)’s Short Read Archive (SRA) database with the accession number SRP126334.

**Retrieval of sequence data from the public database**

In addition to the 16S rRNA amplicon sequence data collected from the produced water samples from Segno and Crossfield oil fields, additional sequence datasets from previous research projects were retrieved from the Short Read Archive (SRA) of the NCBI database and through personal communication. The imported datasets included raw sequence datasets acquired from 16S rRNA amplicon sequencing of 22 produced water samples across the globe. The analyzed samples included six produced water samples from six proximal oil fields in Louisiana (sequenced with Illumina MiSeq), five samples from two oil fields in Xinjiang, China (MiSeq), two samples from two disconnected subseafloor oil fields below the Norwegian Sea (pyrosequencing), and five samples from an off-shore oil field (Halfdan) in the North Sea off the coast of Denmark (MiSeq) (Lewin et al., 2014; Gao et al., 2015; Shelton et al., 2016; Vigneron et al., 2017). The datasets from Louisiana, Xinjiang, and the Halfdan oil field had been deposited in the NCBI SRA datatbase with BioProject accession numbers PRJNA310850, PRJNA25240/PRJNA246768, and PRJNA348365, respectively. The data set from the Norwegian Sea had been deposited in a private database and was obtained through personal communication (Lewin et al., 2014).

***In silico* microbial community analyses**

The raw sequence data were processed using the QIIME pipeline v 1.9.1 with the parameters set to default values (Caporaso et al., 2010). The raw reads were de-multiplexed using the barcode information. Ambiguous reads or reads with quality scores lower than the default cut-off value (Q20) were filtered out and chimeric reads were removed. The filtered reads were then clustered into operational taxonomic units (OTUs) using USEARCH by assigning the reads to the OTUs in the Greengenes v13.8 database (DeSantis et al., 2006) or *de novo* clustering (for reads with no matches in the database) with a cut-off value of 97%. The OTUs were assigned to taxa using the RDP classifier against the Greengenes database (Wang et al., 2007). The OTUs with >0.1 % abundance in at least one of the datasets were subjected to further analyses.

The datasets acquired from the databases targeted different regions of 16S rRNA genes. Therefore, a different OTU assigning strategy was adopted for comparative analyses of these imported datasets. The filtered sequence reads were clustered into Greengenes v13.8 database at a less stringent 95% cut-off to maximize assignment of the reads to the reference sequences with pre-assigned taxonomy. The reads that did not cluster with any reference sequence were discarded.

The beta-diversities between these OTU profiles of the produced water microbial communities were calculated using the *core_diversity_analyses.py* script of the QIIME software package (Caporaso et al., 2010). Three different distance metrics were used for analyses of the beta diversity, including Bray-Curtis dissimilarity indices and weighted and unweighted Unifrac indices (Lozupone and Knight, 2005). These distance metrics were used to construct non-metric multidimensional scaling (NMDS) plots. The OTU network was constructed with *make_otu_network.py* script of the QIIME software package (Caporaso et al., 2010). The co-occurrence analysis was performed using Mothur v1.36.1 package with the sequence data files (in BIOM file format) transformed to .shared files using the *make.shared* script (Schloss et al., 2009). The taxa recovered exclusively in produced water samples from a single oil field site were excluded from the analysis. The Spearman’s rank correlation coefficients between the OTUs were determined using the *otu.association* script in the Mothur software package and a threshold coefficient value of 0.5 was used to screen for the pairs of OTUs with positive correlations. The OTU network and the co-occurrence network were visualized using Cytoscape v3.2.1 (Shannon et al., 2003).

**Supplementary Results**

**Shotgun metagenome sequencing**

Due to the prohibitively low microbial population in the produced water samples, acquisition of sufficient DNA for MiSeq-based shotgun metagenome sequencing (>1 ng DNA needed) was challenging. Acquiring sufficient DNA yield from the produced water samples (up to 1.6 L of liquid volume used) was not possible, save for the CFS sample. The CFS sample (580 mL) was centrifuged at 12,000xg for 30 min and the supernatant was discarded. The pellet was then resuspended with ~3 mL of the remaining produced water. The resulting suspension was centrifuged and the cell pellet was further processed for DNA extraction. The concentration of the extracted DNA was measured using the Qubit fluorometer and a MiSeq sequencing library was constructed using Nextera XT DNA Library Preparation Kit (Illumina) according to the protocol provided by the manufacturer. The quality of the MiSeq sequencing library was confirmed with the bioanalyzer and the library was sequenced using a Miseq benchtop sequencer. The throughput of shotgun metagenome sequencing was insufficient for analyses of functional genes or construction of draft genomes of the major constituents of the microbial community. Thus, the reads from the shotgun metagenome sequencing were used instead for validation of the amplicon sequencing data (Tan et al., 2015). The reads identified as fragments of 16S rRNA genes were extracted from the raw reads data using SortMeRNA v2.0 (Kopylova et al., 2012). These sorted raw reads were processed using the QIIME pipeline as described above. The relative abundance of each taxon was calculated from the abundance of the reads assigned to the taxon as relative to the total number of reads identified as partial fragments of 16S rRNA (Caporaso et al., 2010).

**Microbial community profile in the separator tank**

The samples CFW and CFS both originated from the same oil reservoir in Crossfield, although ~36 h retention in the separator tank (operated at ~50°C) caused a complete change in the structure of the microbial community. The archaeal population was reduced to <0.1% of total microbial population, presumably due to enrichment of the facultatively anaerobic moderately thermophilic bacterial counterparts upon retention in the separator tank as also indicated by the decrease in the Shannon-Wiener diversity index from 6.689 of CFW to 2.370 of CFS (Table S2). The most notable change in the bacterial population was the domination of *Gammaproteobacteria* (64.9%), which constituted just 4.4% of the bacterial population in CFW sample (Fig. 1B). A fast-growing, predominantly mesophilic genus *Pseudomonas* was the dominant taxon constituting 60.8% of the total bacterial population (Table S3). The OTUs affiliated to family *Peptostreptococcacea* of *Clostridia* class and the genus *Bacteroides* of *Bacteroidetes* class were the other dominant taxa in the CFS samples, constituting 24.0% and 7.9% of total community, respectively. All of these taxa were minorities (< 0.02% relative abundance) in the CFW sample (Table S3). *Peptostreptococcaceae* are anaerobic fermentative organisms that carry out acetogenesis in methanogenic environments; members of this family has also been detected in anaerobic (sulfate-reducing) crude oil degrading enrichments (Sherry et al., 2013)

The analysis of short-chain ribosomal RNA gene sequences in the shotgun metagenome dataset confirmed the reliability of the microbial community profile obtained with amplicon sequencing analyses, although it also provided an evidence of the PCR bias, as discrepancy was observed between the constructed community profiles to some degree (Fig. S3). Out of 6,019,522 sequencing reads, 5577 reads were assigned to 16 rRNA genes after quality filtering. A total of 53.3% of the reads assigned to 16S rRNA genes were affiliated to the groups of organisms with >1% abundance in the amplicon sequencing data (97.3% of total reads in the amplicon sequencing data). The relative abundance of *Gammaproteobacteria* appears to be overrepresented due to the PCR bias and the higher alpha diversity (47.4% as compared to 66.1% of amplicon sequencing) and the higher Shannon-Wiener index suggested that the PCR bias in 16S amplicon sequencing skewed the community analysis data towards major populations (Acinas et al., 2005). Nonetheless, 5 of 13 major OTUs (>1% abundance) were represented as major populations in the shotgun metagenome sequencing approach and relatively minor differences in relative abundances of the phylogenetic groups were observed, confirming that the community profiles obtained from amplicon sequencing were reliable (Table S5 and Fig. S3).

**Table S1.** Physicochemical characterization of the produced water samples from Segno and Crossfield oil wells.

| **Sample** | **pH** | **Salinity**  **(mg/L)** | **Acetic acid (mM)** | **Propionic acid (mM)** | **Formic acid (mM)** | **Nitrite**  **(mg/L)** | **Nitrate**  **(mg/L)** | **Phosphate**  **(mg/L)** | **Sulfate**  **(mg/L)** | **Ferrous ion**  **(mg/L)** | **Ferric ion**  **(mg/L)** | **Number of**  **16S rRNA gene copies**  **(copies / mL of sample)** |
| --- | --- | --- | --- | --- | --- | --- | --- | --- | --- | --- | --- | --- |
| SG30 | 5.53 | 976.3 | 2.73 | ND^a^ | ND | ND | ND | ND | 10.5 | 46.5 | 4.8 | 171.9 |
| SG54 | 5.93 | 977.4 | 3.07 | 0.260 | ND | ND | ND | ND | 12.6 | 80.6 | ND | 248.3 |
| SG80 | 8.03 | 976.3 | 10.2 | 1.483 | ND | ND | ND | ND | 3.3 | ND | ND | 461.2 |
| SG85 | 6.23 | 977.1 | 2.78 | ND | ND | ND | ND | ND | 12.7 | 67.2 | ND | 438.8 |
| CFW | 4.70 | 775.4 | 0.79 | ND | 0.403 | ND | ND | ND | 23.7 | 13.9 | ND | 489.2 |
| CFS | 7.33 | 975.7 | 2.06 | ND | ND | ND | ND | 13.0 | 35.32 | 1.5 | 0.3 | 304829.0 |

^a^ ND: below the detection limit

**Table S2.** Summary of 16S rRNA amplicon sequencing of the produced water samples

| **Sample** | **Number of QC reads** | **Archaea (%)** | **Bacteria (%)** | **Shannon’s Index^a^** | **Good’s coverage^a^ (%)** | **Estimated OTUs^a^ (Chao)** | **Number of OTUs** |
| --- | --- | --- | --- | --- | --- | --- | --- |
| SG30 | 45,944 | 33.4 | 66.6 | 5.698 | 99.6 | 1174 | 1072 |
| SG54 | 59,576 | 26.2 | 73.8 | 6.569 | 99.4 | 1587 | 1408 |
| SG80 | 103,164 | 19.4 | 80.6 | 5.202 | 99.3 | 1405 | 1188 |
| SG85 | 47,817 | 23.2 | 76.8 | 6.579 | 99.5 | 1256.3 | 1095 |
| CFW | 77,325 | 18.6 | 81.4 | 6.689 | 99.5 | 1267 | 1086 |
| CFS | 57,554 | 0.1 | 99.9 | 2.370 | 99.8 | 400 | 337 |

^a^ Diversity indices were calculated with the read numbers normalized to 45940.

**Table S4.** Physicochemical characteristics of the produced water samples whose microbial communities were analyzed in this study.

| Sample | Oil field | Temp.  (°C) | pH | Salinity  (mg/L) | Acetic acid (mM) | Propionic acid (mM) | Formic acid (mM) | Nitrite  (mg/L) | Nitrate  (mg/L) | Phosphate  (mg/L) | Sulfate  (mg/L) | Number of eubacterial  16S rRNA gene  (copies / mL of sample) |
| --- | --- | --- | --- | --- | --- | --- | --- | --- | --- | --- | --- | --- |
| SG30 | Segno, TX, USA | 80 - 85 | 5.53 | 976.3 | 2.73 | ND^a^ | ND | ND | ND | ND | 10.5 | 171.9 |
| SG54 |  |  | 5.93 | 977.4 | 3.07 | 0.260 | ND | ND | ND | ND | 12.6 | 248.3 |
| SG80 |  |  | 8.03 | 976.3 | 10.2 | 1.483 | ND | ND | ND | ND | 3.3 | 461.2 |
| SG85 |  |  | 6.23 | 977.1 | 2.78 | ND | ND | ND | ND | ND | 12.7 | 438.8 |
| CFW | Crossfield, AB, Canada | 75 | 4.70 | 775.4 | 0.79 | ND | 0.403 | ND | ND | ND | 23.7 | 489.2 |
| CFS |  | 50 | 7.33 | 975.7 | 2.06 | ND | ND | ND | ND | 13.0 | 35.32 | 304829.0 |
| Lu3065 | Xinjiang, China | 42 | 5.5-6.0 | 10,177 | - | - | - | - | ND | ND | 14.0 | - |
| Lu3096 |  |  | 5.5-6.0 | 9,214 | - | - | - | - | ND | ND | 23.1 | - |
| T90 |  | 22.6 | - | 13,203 | - | - | - | - | - | - | 89.9 | - |
| T95 |  |  | - | 8,997 | - | - | - | - | - | - | 65.6 | - |
| T96 |  |  | - | 9,710 | - | - | - | - | - | - | 165.1 |  |
| CR1 | Lousiana, USA | 28.7 | 7.6 | - | - | - | - | - | - | - | - | - |
| CR2 |  | 25.5 | 7.7 | - | - | - | - | - | - | - | - | - |
| CR3 |  | 24.2 | 7.6 | - | - | - | - | - | - | - | - | - |
| S3 |  | 28.4 | 7.5 | - | - | - | - | - | - | - | - | - |
| TU1 |  | 32.0 | 7.2 | - | - | - | - | - | - | - | - | - |
| O3 |  | 39.8 | 7.1 | - | - | - | - | - | - | - | - | - |
| O4 |  | 41.7 | 6.7 | - | - | - | - | - | - | - | - | - |
| O5 |  | 38.0 | 7.0 | - | - | - | - | - | - | - | - | - |
| O6 |  | 43.2 | 6.7 | - | - | - | - | - | - | - | - | - |
| O7 |  | 41.8 | 7.4 | - | - | - | - | - | - | - | - | - |
| O8 |  | 40.3 | 7.1 | - | - | - | - | - | - | - | - | - |
| O18 |  | 38.4 | 6.9 | - | - | - | - | - | - | - | - | - |
| O26 |  | 33.7 | 6.9 | - | - | - | - | - | - | - | - | - |
| O27 |  | 33.6 | 7.1 | - | - | - | - | - | - | - | - | - |
| O31 |  | 48.5 | 6.9 | - | - | - | - | - | - | - | - | - |
| O32 |  | 40.3 | 6.9 | - | - | - | - | - | - | - | - | - |
| NH1 |  | 44.7 | 6.9 | - | - | - | - | - | - | - | - | - |
| NH2 |  | 45.0 | 6.9 | - | - | - | - | - | - | - | - | - |
| NH3 |  | 38.0 | 6.8 | - | - | - | - | - | - | - | - | - |
| CB1 |  | 24.4 | 6.8 | - | - | - | - | - | - | - | - | - |
| CB3 |  | 29.4 | 6.9 | - | - | - | - | - | - | - | - | - |
| CB4 |  | 28.8 | 7.3 | - | - | - | - | - | - | - | - | - |
| A47 | Norwegian Sea | 86 | - | - | - | - | - | - | - | - | - | - |
| A50 |  | 83 | - | - | - | - | - | - | - | - | - | - |
| HBA-20 | Danish North Sea | 75 | - | - | - | - | - | 0 | - | - | 0 | - |
| HBB-03 |  | 73 | - | 48,315^a^ | - | - | - | 0 | - | - | 4 | - |
| HBB-07 |  | 75 | - | 47,447 ^a^ | - | - | - | 0 | - | - | 3 | - |
| HBB-09 |  | 76 | - | 46,383 ^a^ | - | - | - | 0 | - | - | 1 | - |
| HDA-09 |  | 75 | - | 60,414 ^a^ | - | - | - | 0 | <0 .5 | - | - | - |

^a^The salinities of the Danish North Sea samples were sum of the concentrations of sodium and chloride ions.


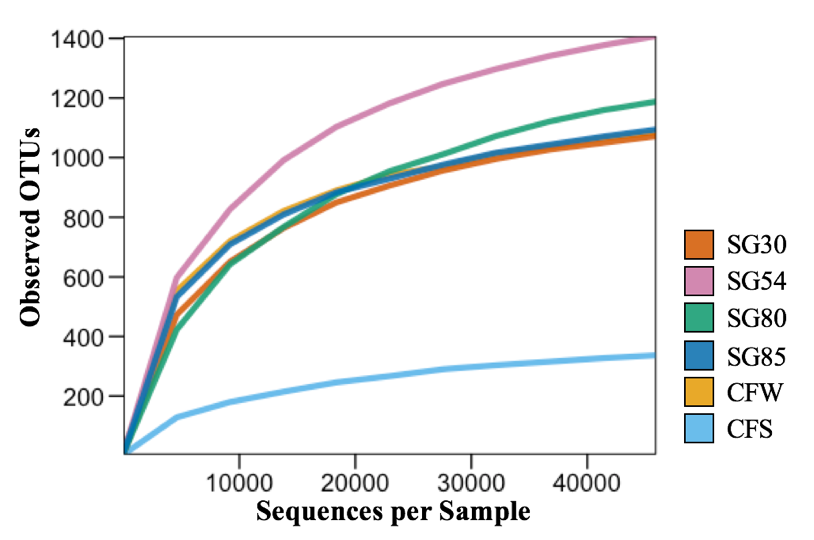


**Figure S1**. Rarefaction curves of the OTUs recovered from the produced water samples.


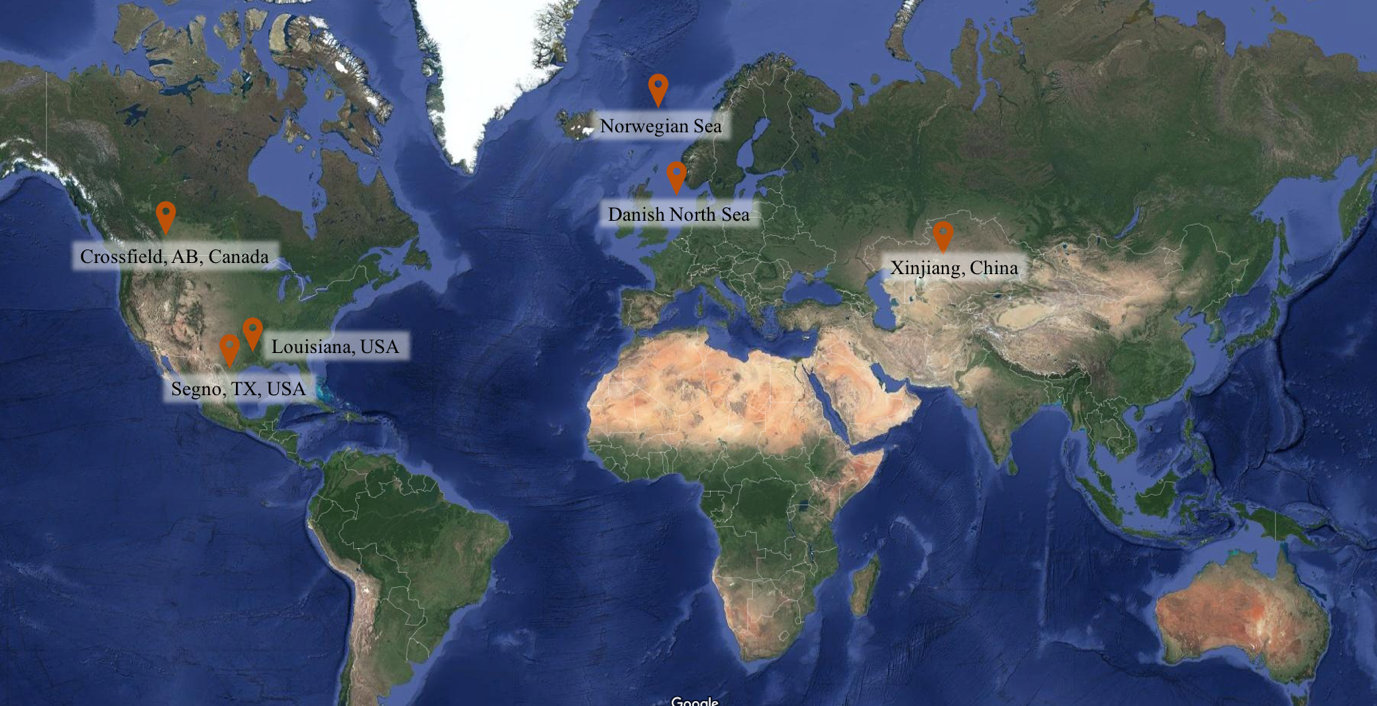


**Figure S2.** Geographical locations of the oil well sites where the produced water samples analyzed in this study were sampled.


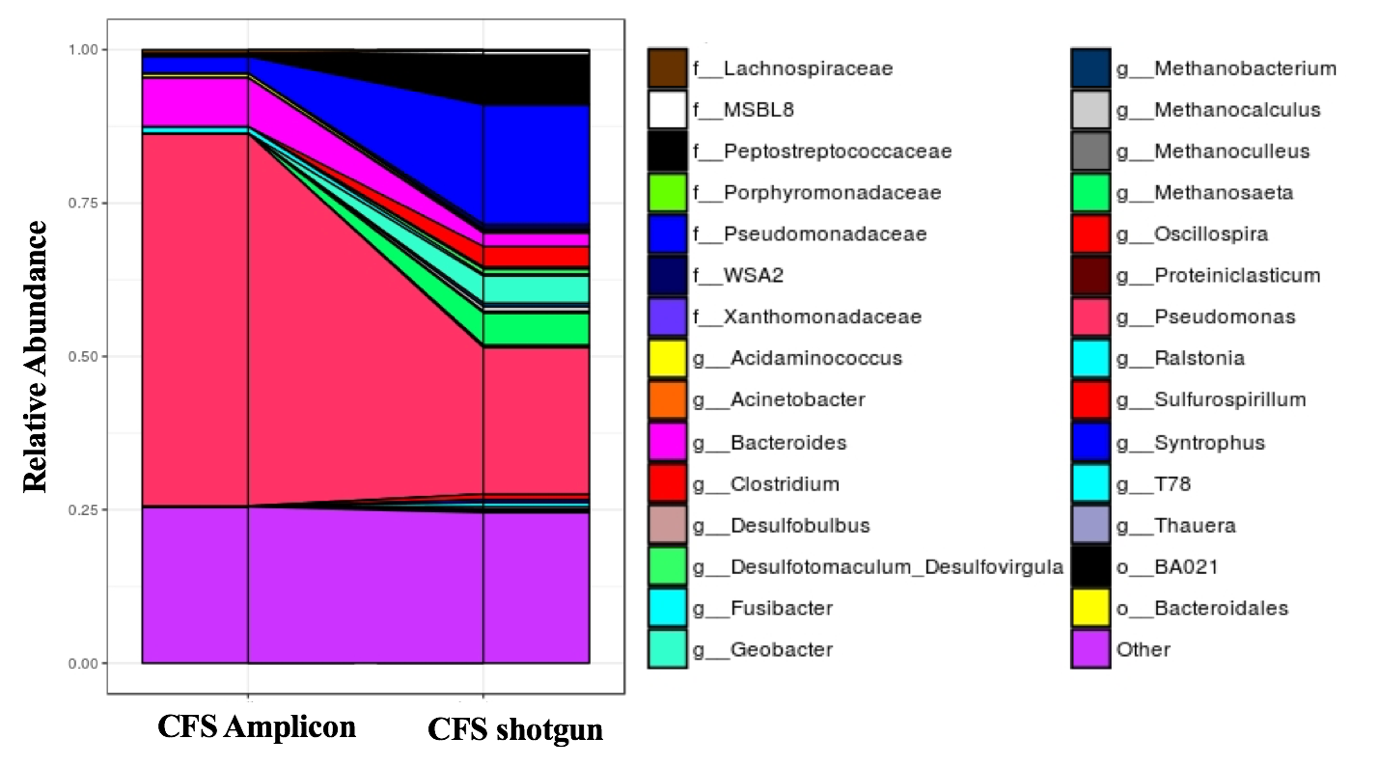


**Figure S3.** Comparison of microbial community profiles of the CFS sample constructed with 16S amplicon sequencing and shotgun metagenome sequencing.

**Reference**

Acinas, S.G., Sarma-Rupavtarm, R., Klepac-Ceraj, V., and Polz, M.F. (2005) PCR-induced sequence artifacts and bias: insights from comparison of two 16S rRNA clone libraries constructed from the same sample. *Appl Environ Microbiol* 71: 8966-8969.

Alain, K., Callac, N., Ciobanu, M.-C., Reynaud, Y., Duthoit, F., and Jebbar, M. (2011) DNA extractions from deep subseafloor sediments: novel cryogenic-mill-based procedure and comparison to existing protocols. *J Microbiol Methods* 87: 355-362.

Caporaso, J.G., Kuczynski, J., Stombaugh, J., Bittinger, K., Bushman, F.D., Costello, E.K. *et al.* (2010) QIIME allows analysis of high-throughput community sequencing data. *Nat Methods* 7: 335-336.

DeSantis, T.Z., Hugenholtz, P., Larsen, N., Rojas, M., Brodie, E.L., Keller, K. *et al.* (2006) Greengenes, a chimera-checked 16S rRNA gene database and workbench compatible with ARB. *Appl Environ Microbiol* 72: 5069-5072.

Gao, P., Tian, H., Li, G., Sun, H., and Ma, T. (2015) Microbial diversity and abundance in the Xinjiang Luliang long‐term water‐flooding petroleum reservoir. *MicrobiologyOpen* 4: 332-342.

Klindworth, A., Pruesse, E., Schweer, T., Peplies, J., Quast, C., Horn, M., and Glöckner, F.O. (2012) Evaluation of general 16S ribosomal RNA gene PCR primers for classical and next-generation sequencing-based diversity studies. *Nucleic Acids Res* 41: e1.

Kopylova, E., Noé, L., and Touzet, H. (2012) SortMeRNA: fast and accurate filtering of ribosomal RNAs in metatranscriptomic data. *Bioinformatics* 28: 3211-3217.

Lewin, A., Johansen, J., Wentzel, A., Kotlar, H.K., Drabløs, F., and Valla, S. (2014) The microbial communities in two apparently physically separated deep subsurface oil reservoirs show extensive DNA sequence similarities. *Environ Microbiol* 16: 545-558.

Lozupone, C., and Knight, R. (2005) UniFrac: a new phylogenetic method for comparing microbial communities. *Appl Environ Microbiol* 71: 8228-8235.

Ritalahti, K.M., Amos, B.K., Sung, Y., Wu, Q., Koenigsberg, S.S., and Löffler, F.E. (2006) Quantitative PCR targeting 16S rRNA and reductive dehalogenase genes simultaneously monitors multiple *Dehalococcoides* strains. *Appl Environ Microbiol* 72: 2765-2774.

Schloss, P.D., Westcott, S.L., Ryabin, T., Hall, J.R., Hartmann, M., Hollister, E.B. *et al.* (2009) Introducing mothur: open-source, platform-independent, community-supported software for describing and comparing microbial communities. *Appl Environ Microbiol* 75.

Shannon, P., Markiel, A., Ozier, O., Baliga, N.S., Wang, J.T., Ramage, D. *et al.* (2003) Cytoscape: a software environment for integrated models of biomolecular interaction networks. *Genome Res* 13: 2498-2504.

Shelton, J.L., Akob, D.M., McIntosh, J.C., Fierer, N., Spear, J.R., Warwick, P.D., and McCray, J.E. (2016) Environmental drivers of differences in microbial community structure in crude oil reservoirs across a methanogenic gradient. *Front Microbiol* 7: 1535.

Sherry, A., Gray, N., Ditchfield, A., Aitken, C., Jones, D., Röling, W. et al. (2013) Anaerobic biodegradation of crude oil under sulphate-reducing conditions leads to only modest enrichment of recognized sulphate-reducing taxa. *Int Biodeter Biodegr* 81: 105-113.

Tan, B., Fowler, S.J., Laban, N.A., Dong, X., Sensen, C.W., Foght, J., and Gieg, L.M. (2015) Comparative analysis of metagenomes from three methanogenic hydrocarbon-degrading enrichment cultures with 41 environmental samples. *ISME J* 9: 2028-2045.

Vigneron, A., Alsop, E.B., Lomans, B.P., Kyrpides, N.C., Head, I.M., and Tsesmetzis, N. (2017) Succession in the petroleum reservoir microbiome through an oil field production lifecycle. *ISME J*. 11: 2141-2154.

Viollier, E., Inglett, P., Hunter, K., Roychoudhury, A., and van Cappellen, P. (2000) The ferrozine method revisited: Fe (II)/Fe (III) determination in natural waters. *Appl Geochem* 15: 785-790.

Wang, Q., Garrity, G.M., Tiedje, J.M., and Cole, J.R. (2007) Naive Bayesian classifier for rapid assignment of rRNA sequences into the new bacterial taxonomy. *Appl Environ Microbiol* 73: 5261-5267.
